# Ginkgolic acid and anacardic acid are specific covalent inhibitors of SARS-CoV-2 cysteine proteases

**DOI:** 10.1186/s13578-021-00564-x

**Published:** 2021-02-28

**Authors:** Zinuo Chen, Qinghua Cui, Laura Cooper, Pin Zhang, Hyun Lee, Zhaoyu Chen, Yanyan Wang, Xiaoyun Liu, Lijun Rong, Ruikun Du

**Affiliations:** 1 College of Pharmacy, Shandong University of Traditional Chinese Medicine, Jinan, 250355 China; 2 Experimental Center, Shandong University of Traditional Chinese Medicine, Jinan, 250355 China; 3 Qingdao Academy of Chinese Medicinal Sciences, Shandong University of Traditional Chinese Medicine, Qingdao, 266122 China; 4 Department of Microbiology and Immunology, College of Medicine, University of Illinois at Chicago, Chicago, IL 60612 USA; 5 Chicago BioSolutions Inc, 2242 W Harrison Street, Chicago, Illinois 60612 United States; 6 Department of Pharmaceutical Sciences, Center for Biomolecular Sciences, College of Pharmacy, Biophysics Core at Research Resources Center, University of Illinois at Chicago, Chicago, IL 60607 USA

**Keywords:** SARS-CoV-2, Natural product, Ginkgolic acid, Anacardic acid, Papain‐like protease

## Abstract

**Background:**

In the urgent campaign to develop therapeutics against SARS-CoV-2, natural products have been an important source of new lead compounds.

**Results:**

We herein identified two natural products, ginkgolic acid and anacardic acid, as inhibitors using a high-throughput screen targeting the SARS-CoV-2 papain-like protease (PL^pro^). Moreover, our study demonstrated that the two hit compounds are dual inhibitors targeting the SARS-CoV-2 3-chymotrypsin-like protease (3CL^pro^) in addition to PL^pro^. A mechanism of action study using enzyme kinetics further characterized the two compounds as irreversible inhibitors against both 3CL^pro^ and PL^pro^. Significantly, both identified compounds inhibit SARS-CoV-2 replication in vitro at nontoxic concentrations.

**Conclusions:**

Our finding provides two novel natural products as promising SARS-CoV-2 antivirals.

## Background

The pandemic of coronavirus disease 2019 (COVID-19), which is caused by the novel severe acute respiratory syndrome coronavirus-2 (SARS-CoV-2), has led to more than 93.81 million confirmed cases and approximately 2.03 million deaths globally (as of January 19, 2021, per the World Health Organization). Vaccines and antivirals are two major countermeasures to combat this viral infection. Encouraging progress has been achieved in vaccine developments, with several vaccine candidates either conditionally approved or in phase III clinical trials [1]. However, escape mutations might occur rapidly [2], and COVID-19 reinfection in the presence of neutralizing antibodies has been observed [3], these observations collectively may compromise the efficacy of vaccines. Regarding drug discovery and development, a repurposed drug remdesivir has been granted authorization by the Food and Drug Administration (FDA) of the United States; however, the efficacy of remdesivir in the treatment of COVID-19 patients is still under debate [4]. Discovery and development of more reliable with high efficacy antivirals are urgent.

SARS-CoV-2 is an enveloped, positive-stranded RNA virus, which belongs to the betacoronavirus genera. In infected cells, translation of the viral RNA initially produce 2 polyproteins, pp1a and pp1ab, which are subsequently cleaved by two viral cysteine proteases, the 3-chymotrypsin-like protease (3CL^pro^) and the papain-like protease (PL^pro^), yielding 16 mature nonstructural proteins [5]. Due to the critical roles of 3CL^pro^ and PL^pro^ during viral polyprotein processing, both 3CL^pro^ and PL^pro^ have been well acknowledged as attractive antiviral drug targets. Up to now, numerous inhibitors directed at 3CL^pro^ have been reported, providing valuable lead compounds for further development [6, 7]. Although there have been less inhibitor-development studies directed at PL^pro^, the structural and functional studies have proven the essential roles of PL^pro^ in viral infection and replication [8]. Beyond viral polyprotein processing, PL^pro^ is also involved in the regulation of innate immune response, by removal of ubiquitin (Ub) and Ub-like protein ISG15 (interferon-induced gene 15) from cellular proteins [9]. A PL^pro^ inhibitor may therefore act in a dual therapeutic manner by both suppressing SARS-CoV-2 replication and promoting antiviral immunity.

We herein optimized a sensitive fluorescence-based high-throughput screen approach to discover SARS-CoV-2 PL^pro^ inhibitors. A library consisting of 1920 natural products was subsequently screened and two compounds ginkgolic acid and anacardic acid were identified to have antiviral potency against SARS-CoV-2.

## Results

### Establishing the fluorescence‐based enzymatic assay for the SARS-CoV-2 PL^pro^

**Fig. 1:**
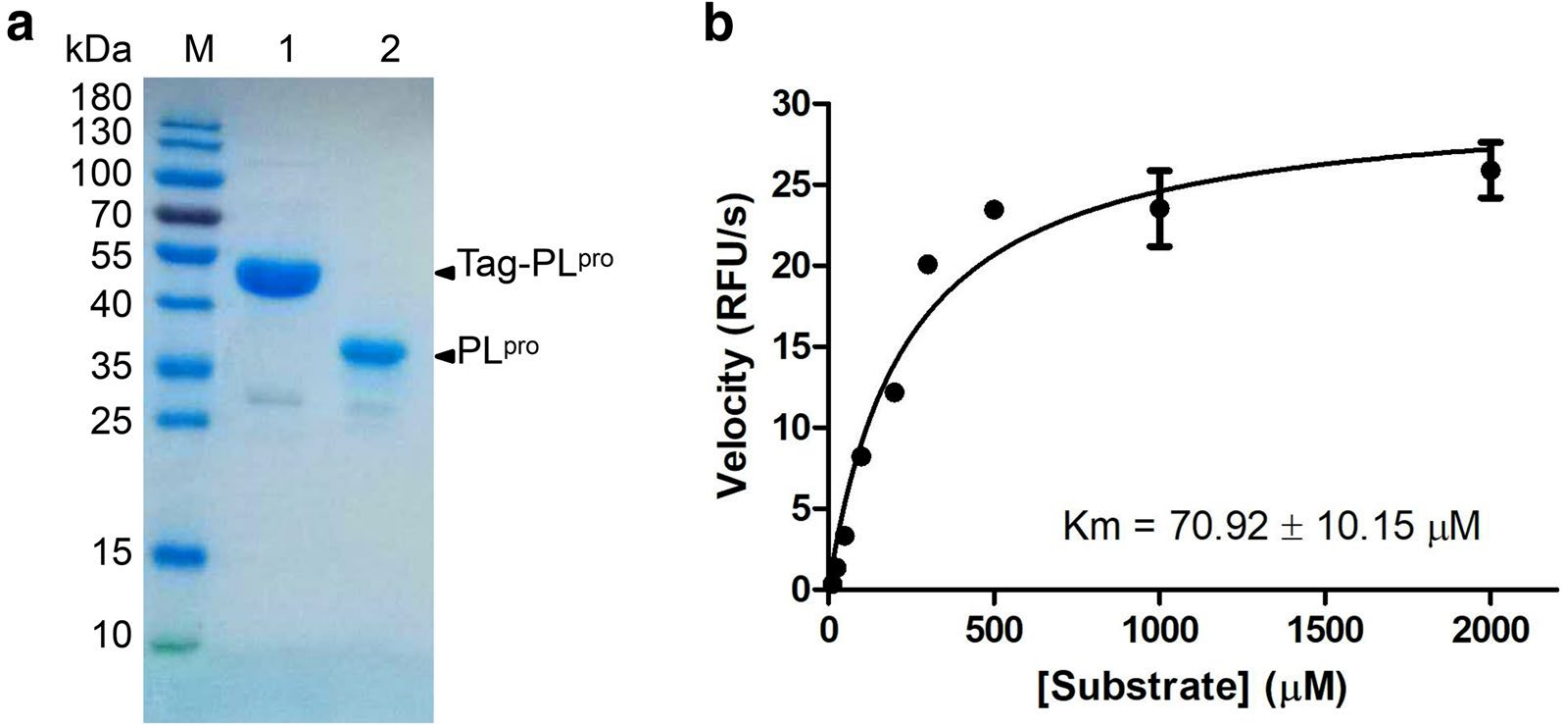
SARS-CoV-2 PL^pro^ expression and characterization. **a** SDS-PAGE of purified PL^pro^. Lane M: protein ladder; lane 1: tagged-PL^pro^; lane 2: authentic PL^pro^. **b** Michaelis–Menten plot of 100 nM PL^pro^ with various concentrations of fluorometric substrate. The best-fit Km = 70.92 ± 10.15 µM

The codon optimized PL^pro^ gene of SARS-CoV-2 was cloned into pET32a(+) vector and expressed in BL21 (DE3) *Escherichia coli.* with Trix-His-S tags fusing to the N-terminus. The PL^pro^ protein was purified with Ni-NTA column to high purity, and the redundant tags were removed by EK digestion prior to activity assays (Fig. 1a). To establish the in vitro enzymatic assay of SARS-CoV-2 PL^pro^, a substrate containing the five C-terminal residues of human ubiquitin with a C-terminal 7-amido-4-methylcoumarin (AMC) group, Z-RLRGG-AMC, was used as previously described [10]. Hydrolysis of the AMC-peptide bond by PL^pro^ can dramatically increase the fluorescence of the AMC moiety. To better characterize the enzymatic property, the Km value of SARS-CoV-2 PL^pro^ was measured. Upon mixture of 100 nM PL^pro^ with various concentrations of fluorometric substrate (0–2 mM), the initial velocity (V_0_) was measured and plotted to substrate concentration. Curve fitting with Michaelis–Menten equation gave the best-fit Km value of 70.92 ± 10.15 µM (Fig. 1b).

### Screen of a natural product library against SARS-CoV-2 PL^pro^

**Fig. 2:**
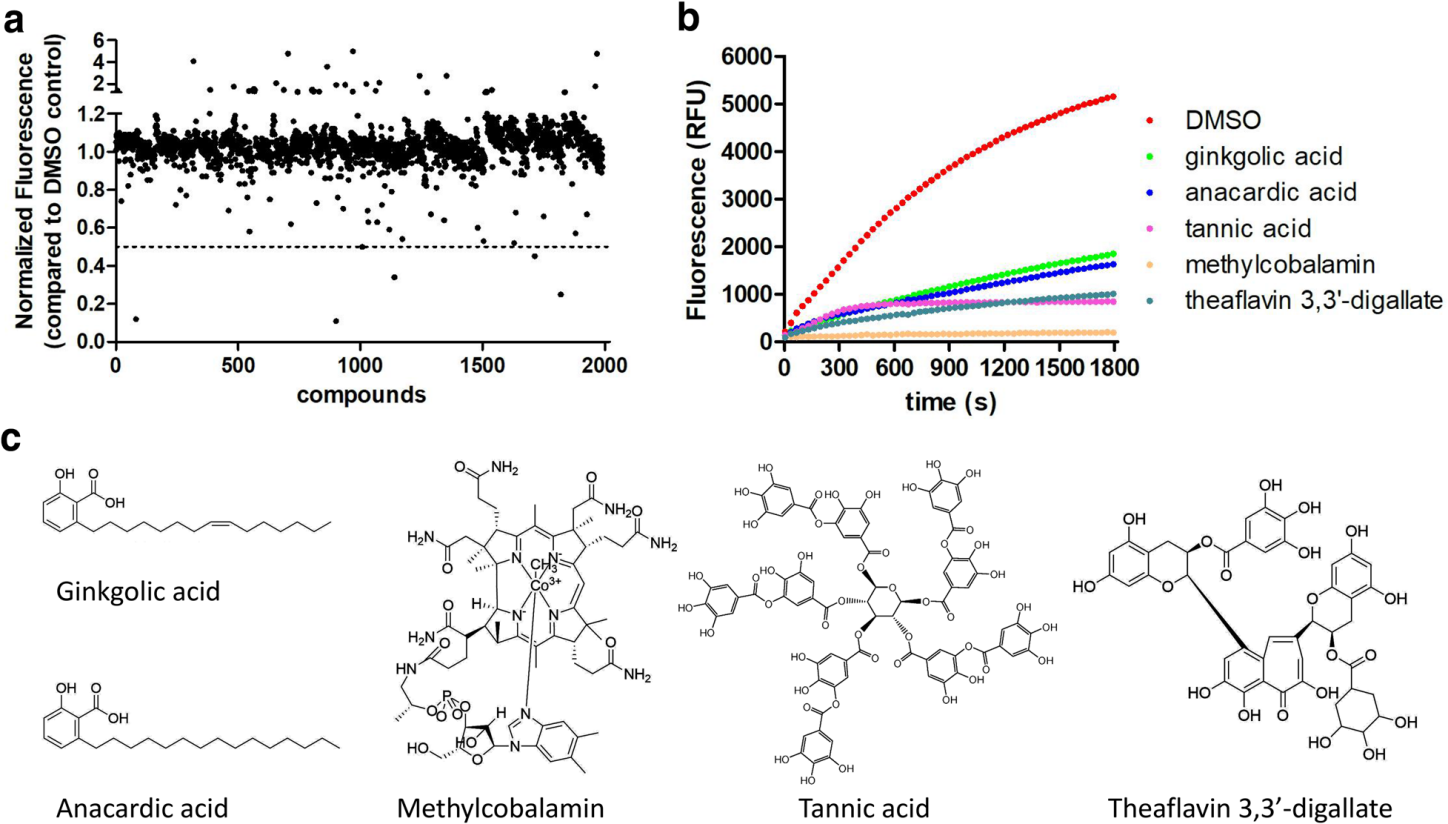
High-throughput screening of a library of natural products against PL^pro^ identifies 5 hit inhibitors. **a** Results from screening of 1920 natural products for inhibition of PL^pro^ activity. The relative fluorescence units (RFUs) at 10 min after reaction initiation were normalized to DMSO control and used to indicate the enzymatic activities. The dashed line indicates the threshold for hit selection (> 50% fluorescence reduction). **b** The original reaction progression curves in presence of DMSO or hit compounds. **c** The structures of the hit compounds

The SARS-CoV-2 PL^pro^ enzymatic assay was next adapted for a high-throughput screening approach, and a library consisting of 1920 natural products was screened to identify potential SARS-CoV-2 inhibitors. The compounds were pre-incubated with 100 nM of PL^pro^ at room temperature for 30 min in reaction buffer containing 4 mM dithiothreitol (DTT) before the addition of 30 µM fluorometric substrate. All compounds were tested at 20 µM. As shown in Fig. 2a, five compounds showed more than 50% inhibition against PL^pro^. Among these hits (Fig. 2b, c), tannic acid, methylcobalamin, and theaflavin 3,3′-digallate have been proposed as potent SARS-CoV-2 inhibitors in previous studies [11–13], while ginkgolic acid and anacardic acid were identified for the first time so far to our knowledge. We therefore focused on these two hits for further analysis.

### Ginkgolic acid and anacardic acid are dual inhibitors targeting both PL^pro^ and 3CL^pro^ of SARS-CoV-2

**Fig. 3:**
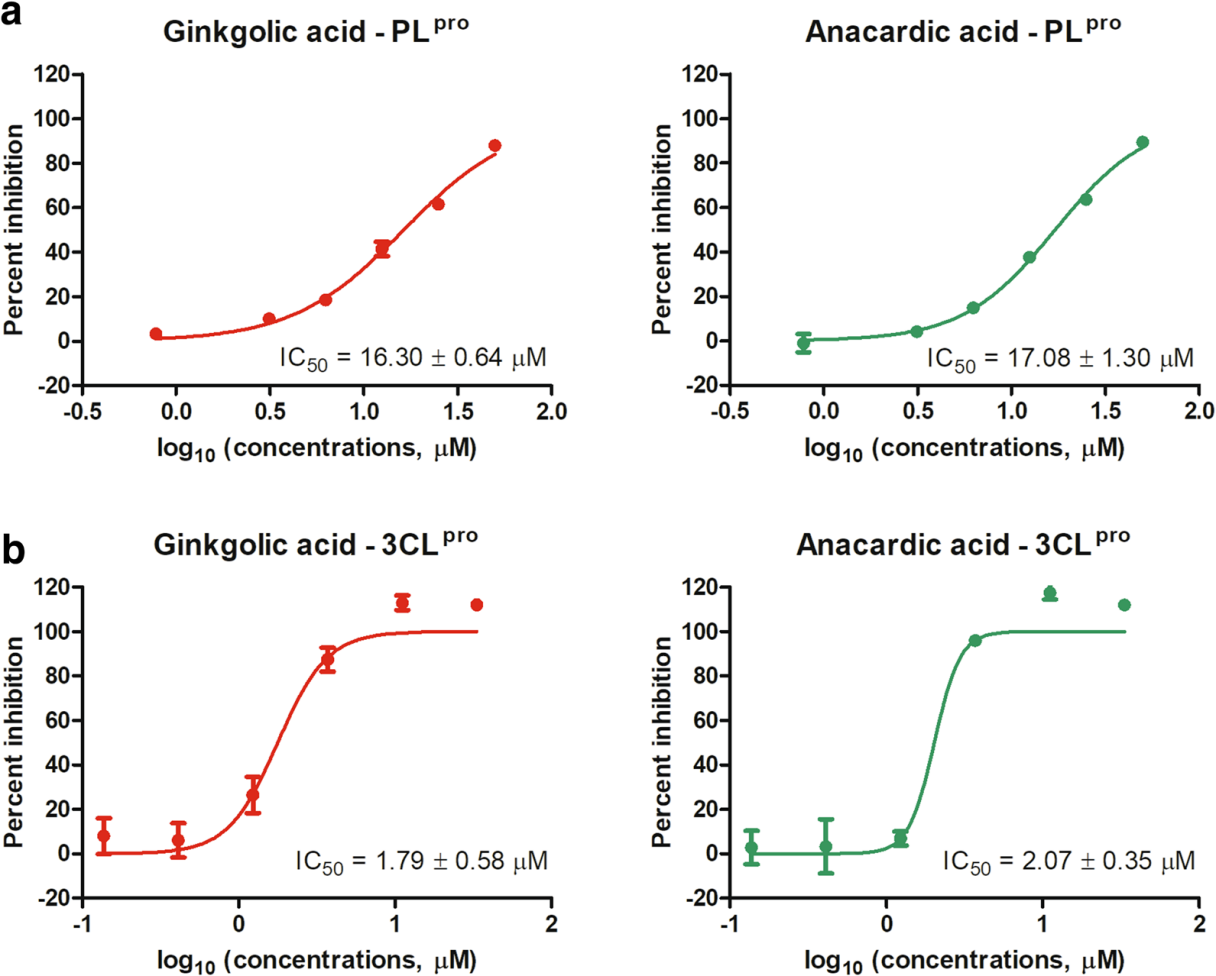
PL^pro^ and 3CL^pro^ enzymatic inhibition assay. The IC_50_ curves of ginkgolic acid and anacardic acid against enzymatic activities of PL^pro^ (**a**) and 3CL^pro^ (**b**) were indicated. For each compound, the IC_50_ value is displayed in the bottom right corner. The data represent mean ± standard deviation (SD) of the triplicate measurements

To validate ginkgolic acid and anacardic acid as PL^pro^ inhibitors, dose response analysis was conducted using an enzymatic inhibition assay. As a result, both ginkgolic acid and anacardic acid dose-dependently inhibited PL^pro^ activity, with IC_50_ values of 16.30 ± 0.64 and 17.08 ± 1.30 µM, respectively (Fig. 3a). Furthermore, neither ginkgolic acid nor anacardic acid interferes with fluorescence detection at tested concentrations (Additional file 1: Figure S1). These results clearly demonstrated ginkgolic acid and anacardic acid as SARS-CoV-2 PL^pro^ inhibitors.

Interestingly, in a parallel screen against SARS-CoV-2 3CL^pro^, ginkgolic acid and anacardic acid were also identified as inhibitors of 3CL^pro^, with IC_50_ values of 1.79 ± 0.58 and 2.07 ± 0.35 µM, respectively (Fig. 3b). Compared to PLpro, 3CLpro is about 10 times sensitive to both ginkgolic acid and anacardic acid. These data suggest that ginkgolic acid and anacardic acid are dual inhibitors against both SARS-CoV-2 3CL^pro^ and PL^pro^.

### Ginkgolic acid and anacardic acid exhibit antiviral activity in vitro

**Fig. 4:**
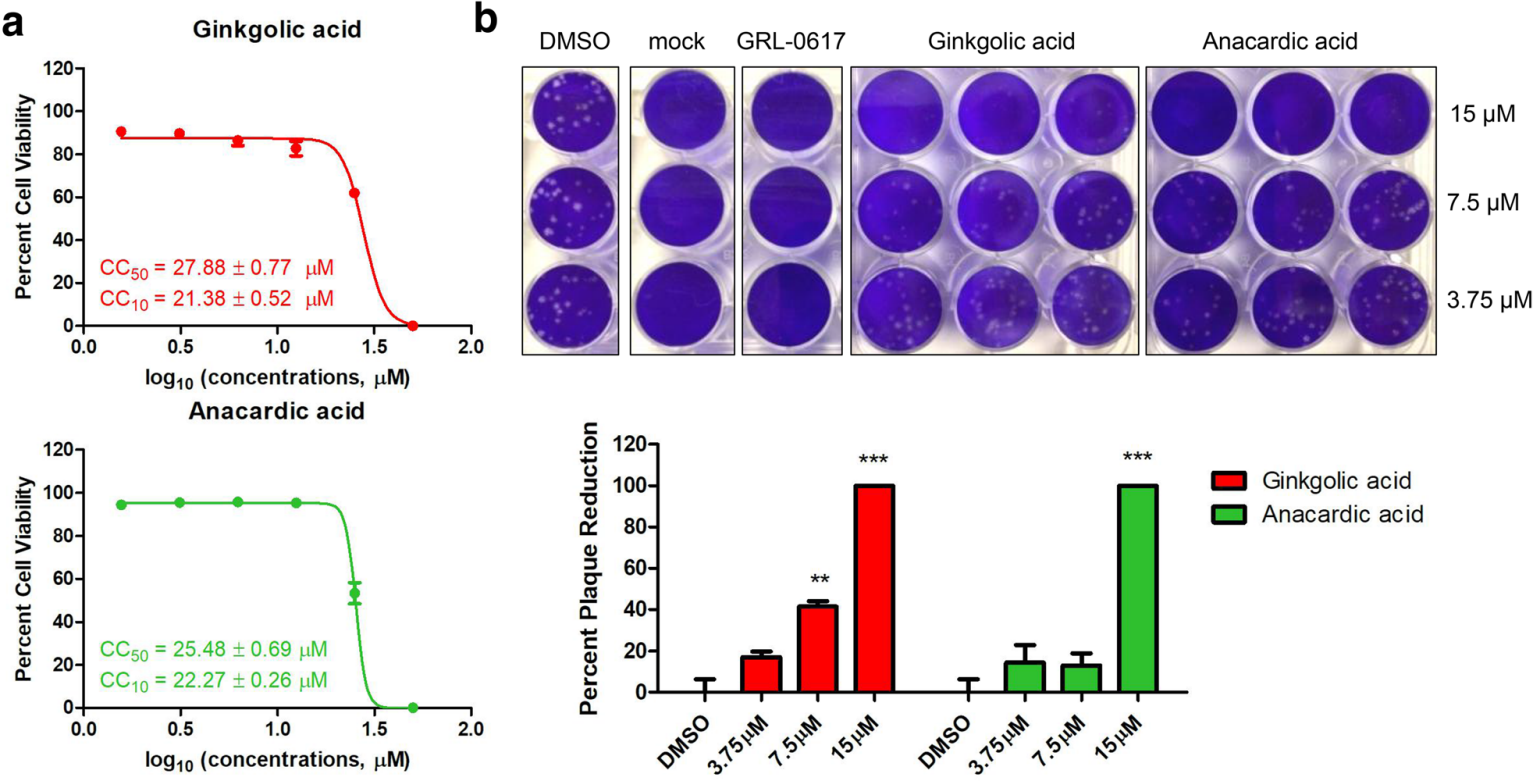
Antiviral determination of ginkgolic acid and anacardic acid against authentic SARS-CoV-2 ***in vitro***. **a** Cytotoxicity curves of ginkgolic acid and anacardic acid in Vero-E6 cells. The CC_50_ and CC_10_ values are displayed in the bottom left corner for each compound. The data represent mean ± standard deviation (SD) of the triplicate measurements. **b** Plaque reduction assay of ginkgolic acid and anacardic acid as well as GRL-0617 (30 µM) against authentic SARS-CoV-2 at indicated concentrations. **p < 0.01,***p < 0.001; student’s t test

To evaluate the antiviral potency of ginkgolic acid and anacardic acid, the two hit inhibitors were subsequently tested against authentic SARS-CoV-2 in vitro. A cytotoxicity assay was performed firstly to determine non-toxic concentrations of ginkgolic acid and anacardic acid. As Fig. 4a shows, the CC_50_ values of ginkgolic acid and anacardic acid to Vero-E6 cells are 27.88 ± 0.77 and 25.48 ± 0.69 µM, respectively. No obvious toxicity (> 90% cell viability) was observed at 20 µM for both ginkgolic acid and anacardic acid.

When subjected to a viral plaque reduction assay with authentic SARS-CoV-2, both ginkgolic acid and anacardic acid significantly reduced virus plaque formation at a concentration of 15 µM (Fig. 4b), suggesting that the two hit inhibitors can block SARS-CoV-2 replication at non-toxic concentrations. Of note, ginkgolic acid showed 42% inhibition at 7.5 µM with estimated EC_50_ value of 8.3 ± 0.03 µM compared to 13% inhibition at 7.5 µM with estimated EC_50_ value of 9.0 ± 2.5 µM for anacardic acid, indicating a higher antiviral potency (Fig. 4b).

### Ginkgolic acid is an irreversible inhibitor against 3CL^pro^ and PL^pro^

**Fig. 5:**
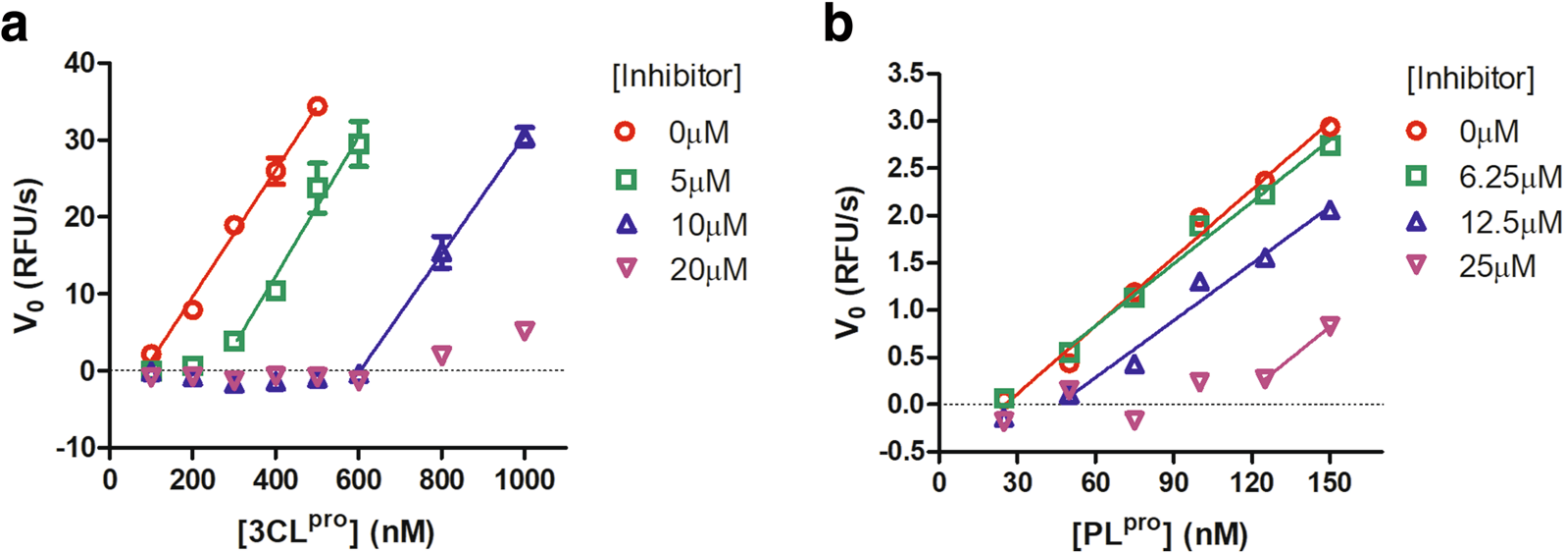
Graphical determination of the type of enzymatic inhibition. Relationship of the enzymatic activity of SARS-CoV-2 3CL^pro^ (**a**) and PL^pro^ (**b**) as function of enzyme concentrations at different concentrations of ginkgolic acid

Given the high structural similarity of ginkgolic acid and anacardic acid, we focused on ginkgolic acid to further elucidate its mechanism of action. The proteolytic activity of the 3CL^pro^ was firstly measured using a series of enzyme concentrations and at various inhibitor concentrations. The substrate concentrations were held constant at sub-saturation levels. At each concentration of ginkgolic acid, the initial reaction velocities were plotted with the concentrations of 3CL^pro^, and the data was fitted using linear regression analysis. As shown in Fig. 5a, as the concentration of ginkgolic acid increases, the best-fit line shifts to right, without altering the slope significantly. Similar results were obtained for ginkgolic acid against PL^pro^ (Fig. 5b). This phenomenon clearly implies that ginkgolic acid act as an irreversible inhibitor against both SARS-CoV-2 cysteine proteases.

### In silico docking

**Fig. 6:**
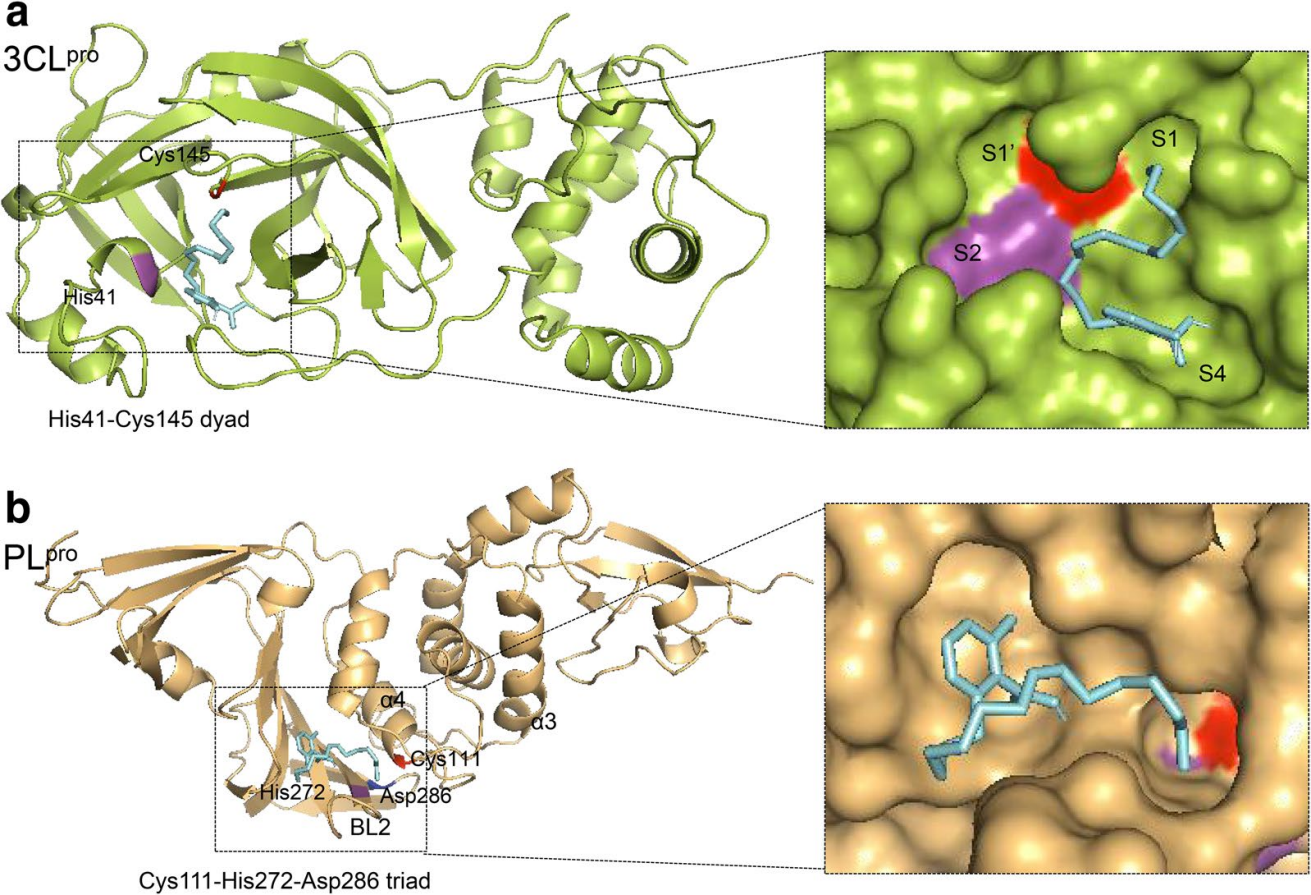
Predicted binding modes of ginkgolic acid to SARS-CoV-2 3CL^pro^ (**a**) and PL^pro^ (**b**). The 3CL^pro^ (PDB ID: 6m2n) and PL^pro^ (PDB code: 6WX4) are shown in green and orange cartoon respectively. The ginkgolic acid is shown in cyan sticks. The enlarged views represent ginkgolic acid in the active site with a view of the molecular surface of SARS-CoV-2 3CL^pro^ (**a**) and PL^pro^ (**b**). The His41-Cys145 dyad of 3CL^pro^ and Cys111-His272-Asp286 triad of PL^pro^ were indicated

To better deduce the binding modes involved in the interaction of ginkgolic acid with SARS-CoV-2 cysteine proteases, an in silico docking study was performed using AutoDock Vina software. As a result, the substrate binding pockets of both 3CL^pro^ and PL^pro^ can be occupied by ginkgolic acid, with predicted affinity of − 5.3 and − 4.9 kcal/mol respectively. The binding mode of ginkgolic acid to 3CL^pro^ is described in Fig. 6a. Among the four sites (S1′, S1, S2 and S4) within the highly conserved active cavity of 3CL^pro^, ginkgolic acid mainly targets to S1 and S4 [6]. On the other hand, as depicted in Fig. 6b, ginkgolic acid accommodates in the substrate cleft formed between the BL2 loop and the α3-α4 loop of PL^pro^, which is almost identical to the mode of GRL-0617 [8].

## Discussion

The development of cysteine protease inhibitors is usually plagued with either notable toxicities or lack of specificity due to covalent modification of untargeted cysteine residues [10]. It was argued that the scientific community should be aware of these nonspecific effects, which could be ruled out by addition of reducing reagents in the enzymatic buffer [14]. In the present study, we identified ginkgolic acid and anacardic acid as novel SARS-CoV-2 cysteine protease inhibitors. Upon addition of reducing reagent in the 3CL^pro^ enzymatic assay, no significant IC_50_ shift was observed (Additional file 2: Figure S2), suggesting the specificity of ginkgolic acid and anacardic acid as cysteine protease inhibitors [14].

Given the fact that ginkgolic acid and anacardic acid inhibit both 3CL^pro^ and PL^pro^ of SARS-CoV-2, one may be concerned that the two inhibitors are potent broadly cysteine protease inhibitors [15]. Although this usually means the compounds are toxic to host cells by targeting cellular cysteine proteases, our study demonstrated the antiviral activities of ginkgolic acid and anacardic acid against authentic SARS-CoV-2 at non-toxic concentrations, suggesting the two compounds deserve further development as novel potential COVID-19 therapies.

Based on our studies, ginkgolic acid act as an irrivesible inhibitor against both PL^pro^ and 3CL^pro^, suggesting it a covalent inhibitor. As described above, the specificity is critical during development of a covalent cysteine protease inhibitor, which could be achieved by rational design. A two-step mechanism of action is usually adopted by those specific covalent inhibitors, such as peptidic inhibitors of SARS-CoV 3CL^pro^, N3 and GC376 [7, 16]. First, the inhibitor may competitively bind to the substrate binding cavity of SARS-CoV-2 cysteine proteases, bringing a warhead to a close proximity to catalytic cysteine. Second, a covalent bond would form at slower velocity between the inhibitor and target cysteine, conferring an irreversible inhibition. We speculate ginkgolic acid and anacardic acid act in the similar two-step mechanism of action as specific covalent inhibitors.

## Conclusions

We herein report ginkgolic acid and anacardic acid as two novel SARS-CoV-2 inhibitors dually targeting 3CL^pro^ and PL^pro^. These two inhibitors are valuable to be developed as a new class of specific covalent cysteine protease inhibitors.

### Methods

#### Reagents and viruses

The library containing 1920 natural products was purchased from MedChemExpress (MCE; Monmouth Junction, NJ, USA). The clinical isolate of SARS-CoV-2 (SARS-CoV-2, Isolate USA-WA1/2020) was obtained from BEI Resources and manipulated in BSL3 containment at University of Illinois at Chicago (Chicago, IL).

### Expression and purification of SARS-CoV-2 PL ^pro^ from *Escherichia coli*

The PL^pro^ gene was amplified from the codon-optimized nsp3 template (kindly gifted by Dr. Shengce Tao from Shanghai Jiaotong University) using primers PL^pro^-forward: 5′-GGCCGGATCCGGTCACCGGTTTAATGGTGGTG-3′ and PL^pro^-reverse: 5′-GGCCAAGCTCTATCGTGAGGTTCGTACCATCAAG-3′, and cloned into the pET-32a(+) expression vector followed by transformation into BL21 (DE3) competent cells. The expression plasmid was constructed such that PL^pro^ carried N-terminal Trix-His6-S tag followed by an enterokinase cleavage site. Correct clones were verified by DNA sequencing. The protein expression was performed as previously described [17].

The purification of recombinant PL^pro^ was carried out using the BeyoGold His-tag protein purification kit (Beyotime, Shanghai, China) according to manufacturer’s manual. The N-terminal tags were removed by recombinant EK digestion prior to enzymatic assay.

### Enzymatic assay of SARS-CoV-2 PL^pro^

A fluorometric peptide Z-Arg-Leu-Arg-Gly-Gly-AMC (Z-RLRGG-AMC) was purchased from NJpeptide and used as substrate in PL^pro^ enzymatic assay as described previously [10]. Upon cleavage by PL^pro^, the fluorescence of the AMC moiety dramatically increases, accurately reflecting the conversion. For PL^pro^ inhibition assay, varying concentrations of inhibitor were mixed with 100 nM PL^pro^ in 90 µL reaction buffer, containing 20 mM Tris-buffer (pH 8.0) and 4 mM DTT, and incubated for 30 min. Reactions were initiated by adding 10 µL Z-RLRGG-AMC with a final concentration of 30 µM. The fluorescence signal was immediately measured every 30 s for 60 min using a Bio-Tek Synergy LX plate reader with filters for excitation at 360/20 nm and emission at 460/20 nm. The initial reaction velocities (V_0_) of reactions were calculated to indicate the enzymatic activities. Three independent experiments were performed and IC_50_ curves were analyzed using four-parameter logistic regression in GraphPad Prism software. Note that the relative fluorescence units (RFUs) at 10 min after reaction initiation were used to indicate the enzymatic activities for high-throughput screen.

For mechanism of action study, various concentrations of ginkgolic acid were mixed and incubated with increasing concentrations of PL^pro^ (25–150 nM) in 90 µL reaction buffer for 30 min. Reactions were initiated by adding 10 µL substrate with a final concentration of 30 µM. At each concentration of ginkgolic acid, the V_0_s were plotted with the concentrations of PL^pro^, and the data was fitted using linear regression analysis in GraphPad Prism software.

### Enzymatic assay of SARS-CoV-2 3CL^pro^

The SARS-CoV-2 3CL^pro^ was prokaryotic expressed and purified as previously described with slight modification [7]. For enzymatic inhibition assay, the recombinant 3CL^pro^ (250 nM at a final concentration) was incubated with increasing concentrations of each compound in 90 µL reaction buffer (50 mM Tris–HCl, pH 7.3, 1 mM EDTA, 4mM DTT) for 30 min [6]. The reaction was initiated by adding 10 µL FRET-based peptidic substrate (Dabcyl-KTSAVLQ/SGFRKME-Edans) with a final concentration of 50 µM. The fluorescence signal was immediately measured every 20 s for 30 min using a Bio-Tek Synergy4 plate reader with filters for excitation at 336/20 nm and emission at 490/20 nm. The V_0_ of reactions were calculated to indicate the enzymatic activities. Three independent experiments were performed and IC_50_ curves were analyzed using four-parameter logistic regression in GraphPad Prism software.

For mechanism of action study, various concentrations of ginkgolic acid were mixed and incubated with increasing concentrations of 3CL^pro^ (100–1000 nM) in 90 µL reaction buffer for 30 min. Reactions were initiated by adding 10 µL substrate with a final concentration of 50 µM. At each concentration of ginkgolic acid, the V_0_s were plotted with the concentrations of 3CL^pro^, and the data was fitted using linear regression analysis in GraphPad Prism software.

### Antiviral determination

To examine the antiviral activity against authentic SARS-CoV-2 (Isolate USA-WA1/2020, from BEI Resources), a plaque reduction assay was conducted. Briefly, Vero-E6 monolayers grown in 12 well plates were pre-treated with various concentrations of test compound for 1 h, followed by infection with SARS-CoV-2 (MOI of 0.0001) in the presence of test compounds. DMSO and a known PL^pro^ inhibitor GRL-0617 (30 µM) were used as negative and positive controls respectively. After 1-h incubation, the medium was replaced with fresh MEM containing 1.25% Avicel and the test compounds, and the plates were incubated for another 48 h at 37 °C and 5% CO_2_. Then cells were fixed with 10% formalin and stained with 1 % crystal violet to visualize plaques. Three independent experiments were performed, and all data was normalized to virus alone. All experiments were performed in a Biosafety level 3 facility.

### Cytotoxicity assay

To estimate the cytotoxicity of test compounds on VERO-E6 cells, Cell-Titer Glo® luminescent cell viability assay (Promega) was performed according to the manufacturer’s instruction. Briefly, the drugs were 2-fold serially diluted starting at 50µM in phenol red free DMEM and were incubated with cells for 48 h at 37 °C and 5% CO_2_. The half of the cytotoxic concentration (CC_50_) values were calculated from the percentages of cells whose viability was inhibited by test compounds at various concentrations.

### In silico docking

For the molecular docking simulations, the structures of 3CL^pro^ (PDB code: 6m2n) and PLpro (PDB code: 6WX4) from SARS-CoV-2 were used. Before docking, polar hydrogen atoms were added to the target protein using autodock tools. Docking was performed using a grid box covering the entire structure by AutoDock Vina software [18]. After docking, the conformation of the compound was analyzed, and the 3D models were viewed with PyMOL.

## Supplementary Information

**Additional file 1: Figure S1.** Ginkgolic acid and anacardic acid don’t interfere with fluorescence detection.

**Additional file 2: Figure S2.** The IC_50_ curves of ginkgolic acid and anacardic acid against enzymatic activities of 3CL^pro^ in absence or presence of 4 mM DTT.

## Data Availability

All data generated or analyzed during this study are included in this article.

## Abbreviations

COVID-19: Coronavirus disease 2019
SARS-CoV-2: Severe acute respiratory syndrome coronavirus-2
3CL^pro^: 3-Chymotrypsin-like protease
PL^pro^: Papain-like protease
Ub: Ubiquitin
AMC: 7-Amido-4-methylcoumarin
DTT: Dithiothreitol
RFU: Relative fluorescence units
