# Supplementary material for: Ginkgolic acid and anacardic acid are specific covalent inhibitors of SARS-CoV-2 cysteine proteases
Source: Cell Biosci. 2021 Feb 28;11:45. doi: 10.1186/s13578-021-00564-x (PMC7914117; doi:10.1186/s13578-021-00564-x)
Supplement: Supplementary file 1 — Additional file 1: Figure S1. Ginkgolic acid and anacardic acid don’t interfere with fluorescence detection. [file 13578_2021_564_MOESM1_ESM.docx]

Additional File 1：

Figure S1. Ginkgolic acid and anacardic acid don’t interfere with fluorescence detection.


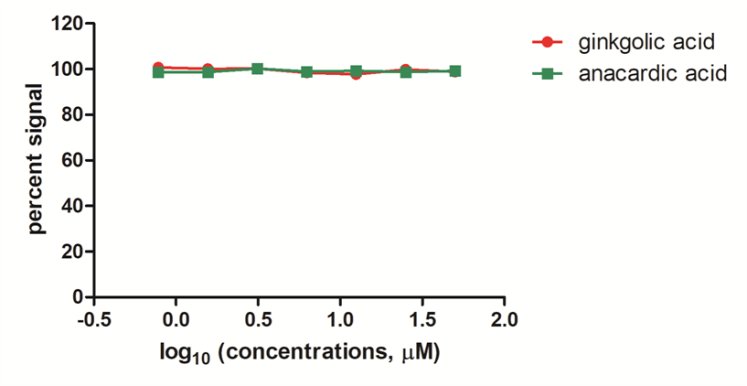


Figure S1. Ginkgolic acid and anacardic acid don’t interfere with fluorescence detection. The recombinant PL^pro^ (100 nM at a final concentration) was incubated with fluorometric peptidic substrate (Z-RLRGG-AMC,30 μM at final concentration) in 90 μL reaction buffer (20 mM Tris–HCl, pH 8.0, 4mM DTT), and incubated at room temperature for 1h, generating a constant fluorescence signal. Increasing concentrations of hit inhibitor in volume of 10 μL were then added and mixed before subjecting to detect the fluorescence signal using a Bio-Tek Synergy LX plate reader with filters for excitation at 360/20 nm and emission at 460/20 nm.
