# Supplementary material for: Ginkgolic acid and anacardic acid are specific covalent inhibitors of SARS-CoV-2 cysteine proteases
Source: Cell Biosci. 2021 Feb 28;11:45. doi: 10.1186/s13578-021-00564-x (PMC7914117; doi:10.1186/s13578-021-00564-x)
Supplement: Supplementary file 2 — Additional file 2: Figure S2. The IC50 curves of ginkgolic acid and anacardic acid against enzymatic activities of 3CLpro in absence or presence of 4 mM DTT. [file 13578_2021_564_MOESM2_ESM.docx]

Additional File 2：

Figure S2. The IC_50_ curves of ginkgolic acid and anacardic acid against enzymatic activities of 3CL^pro^ in presence or absence of 4mM DTT.


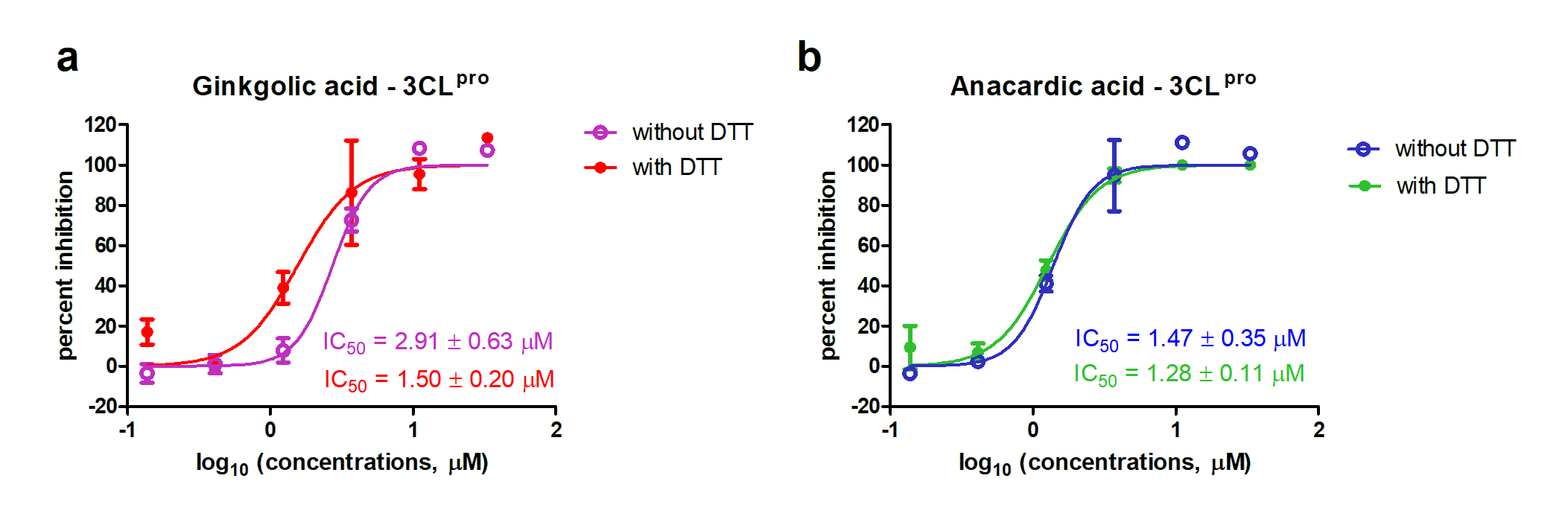


Figure S2. The IC_50_ curves of ginkgolic acid and anacardic acid against enzymatic activities of 3CL^pro^ in presence or absence of 4mM DTT. The IC_50_ values are displayed in the bottom right corner. The data represent mean ± standard deviation (SD) of the triplicate measurements.
